# A confirmatory factor analysis of the Iranian version of the interpersonal communication skills scale among healthcare professionals

**DOI:** 10.1186/s12909-023-04878-x

**Published:** 2023-11-20

**Authors:** Arash Salahshouri, Sheida Fathi, Mostafa Jiba, Hashem Mohamadian, Jafar Kordzanganeh

**Affiliations:** 1 Health Education & Promotion Department, School of Health, Ahvaz Jundishapur University of Medical Sciences, Ahvaz, Iran; 2 Master of Science in Health Education & Promotion, Ahvaz West Health Center, Ahvaz Jundishapur University of Medical Sciences, Ahvaz, Iran; 3 Public Health Department, School of Health, Hamadan University of Medical Sciences, Hamadan, Iran; 4 Social Science Department, Payame Noor University, Tehran, Iran

**Keywords:** Interpersonal communication skills, Healthcare professionals, Comprehensive health centers, Importance performance map analysis, Confirmatory factor analysis

## Abstract

**Background:**

Identifying healthcare professionals’ (HCPs) communication skills is crucial to improving patient outcomes. Iranian HCPs’ interpersonal communication skills (ICS) were validated using a culturally appropriate and indigenous scale.

**Materials and methods:**

In November and December 2021, convenience sampling was used to collect data from 170 HCPs. Seven factors were covered by the questionnaire, which consisted of 30 items. In order to validate the scale, first- and second-order confirmatory factor analyses (CFA) were performed. Various indices were used during the CFA, including Goodness of Fit Index (GFI), Adjusted Goodness of Fit (AGFI), Comparative Fit Index (CFI), Normed Fit Index (NFI), Standardized Root Mean Square Residual (SRMR), and Root Mean Square Error of Approximation (RMSEA). The Fornell-Larcker Criterion was used to assess discriminant validity. We analyzed the data in Lisrel 8.8 and SmartPLS 3.2.8.

**Results:**

According to the Q2-index obtained from the blindfold test, the model had 44% predictive power. First-order CFA results showed acceptable indices (χ2 = 767.17; DF = 375; CFI = 0.98; GFI = 0.82; AGFI = 0.80; NFI = 0.97; SRMR = 0.22; RMSEA = 0.068). Furthermore, the second-order measurement model demonstrated adequate and desirable fit indices (χ2 = 797.24; DF = 381; CFI = 0.98; GFI = 0.82; AGFI = 0.78; NFI = 0.97; SRMR = 0.059; RMSEA = 0.068). General and listening skills were ranked highest in the importance-performance map analysis (IPMA).

**Conclusion:**

HCPs could benefit from this scale as it can assist them in developing ICS. It is recommended that skills training programs be replicated among different populations to evaluate their effectiveness.

## Introduction

To deliver high-quality care, healthcare professionals (HCPs) need effective interpersonal communication skills (EICS) [1]. Nursing and medical professions require proficiency in interpersonal communication skills (ICS), which are also known as EICS. The EICS plays a crucial role in facilitating communication in the healthcare setting, where communication failures can lead to severe consequences [2]. EICS is not merely an attribute; it is the cornerstone of clinical competence and commendable medical practices. HCPs with EICS significantly increase the likelihood of successful patient outcomes while simultaneously reducing the risk of patient complaints and legal disputes.

The COVID-19 pandemic has highlighted the significance of ICS in a way never seen before [3]. Understandably HCPs are anxious about patients’ health during this crisis [4]. The lack of proper training and preparation in ICS for numerous HCPs is a troubling issue that cannot be ignored due to its undeniable significance. Patient care can be adversely affected by insufficient ICS training. In order to effectively communicate about health, it is crucial to develop strategies that are clear, comprehensive, and readily accessible. These strategies aim to ensure that information is easily understood and can be put into practice by society as a whole [5]. Through ICS, patients can retain valuable medical information but also improve their quality of life.

A robust health education system can only function effectively with a proficient healthcare workforce [6]. A city’s overall health and well-being can be a better indication of its sustainable development [7]. A lack of EICS among HCPs can lead to severe errors for patients [8]. To deliver quality healthcare, HCPs must build and foster trust [9]. EICS can be significantly hindered by a lack of trust, which can then hinder treatment overall. HCPs must show compassion when interacting with patients, dedicating enough time to truly understand their needs, actively listening to their worries, and offering expert advice and assistance to resolve them [10] effectively.

To study ICS within the healthcare setting, we adopt the transactional model of interpersonal communication [11]. Various contexts, including personal and professional relationships, as well as educational settings, can benefit from this model. Barnlund founded it. He describes communication as a dynamic, two-way process involving the simultaneous sending and receiving of messages [12]. It focuses on verbal and non-verbal communication, active listening, empathy, emotional regulation, cultural competence, and confirming patient understanding. Improved patient satisfaction, better adherence to treatment plans, stronger provider-patient relationships, and greater health literacy are all dependent on these factors.

HCPs’ critical role in encouraging healthy behavior and implementing customized interventions is vital for their clients [13]. HCPs must possess sufficient health literacy to empower patients and promote healthy behavior change [14]. Thus, HCPs should regularly assess their ICS and receive appropriate training when necessary to ensure that they are up-to-date. For making effective healthcare decisions and formulating policies, it is essential to have precise measurements. A valid EICS assessment tool is essential to this process. Various tools have been designed for specific purposes within the realm of public health education [15–18].

Also, Vakili et al. [19] and Ghasemi and Rasekh [20] suggested other groups of HCPs to develop instruments that help them improve listening skills, ability to communicate verbally and nonverbally, assertiveness, communication understanding, and emotional regulation. Several studies identified various factors [21–23], and in order to ensure its reliability and validity, the scale needs to be evaluated using various methodologies, including confirmatory factor analysis (CFA). A CFA enhances the quality and rigor of research by confirming the validity and reliability of measurement instruments, testing theoretical models, and providing insights for refining constructs as appropriate. A literature review was performed to define the theoretical framework of validity in the context of CFA [24]. CFA assesses construct validity through various fit indices [25], including convergent validity, discriminant validity, and model fit. Convergent validity was assessed using factor loadings, construct reliability (CR), and average extracted variable (AVE) values 0.7 or higher. Discriminant validity was assessed using cross-loadings and the fornell-larcker criterion, with the square root of AVE of each construct larger than its highest correlation with any other construct. Model fit was determined using various fit indices, such as the goodness-of-fit index (GFI), the adjusted goodness-of-fit index (AGFI), the comparative fit index (CFI), the relative/normed fit index (NFI) (more than 0.8), root-mean-square error of approximation (RMSEA), and standardized root mean square residual (SRMR) (less than 0.08). A non-significant chi-square value suggests a good fit but is sensitive to sample size.

In this regard, a reliable tool tailored to Iranian HCPs needs to be developed and validated. Through the use of theoretical foundations, our goal is to provide a deeper understanding of the profound effect that ICS has on healthcare services, particularly within Iran’s distinctive cultural context. Therefore, through the development and validation of the EICS, we have the potential to make a significant impact on communication and enable HCPs to promote healthy behaviors in their communities successfully.

## Methods

### Study design and setting

The research was carried out in Urban and Rural Comprehensive Healthcare Centers (CHCs) under the supervision of Ahvaz Jundishapur University of Medical Sciences (AJUMS).

### Study subjects

Participants were included in the study once they gave informed consent. A total of 180 HCPs provided voluntary services to clients as part of this study. In order to assess participant eligibility, we set up inclusion and exclusion criteria. To be eligible for participation, HCPs had to have three years of experience and affiliation with a rural or urban CHCs. In addition, those without a university degree or without answers to the questionnaire were excluded.

### Sampling method and sample size

A minimum sample size is essential for structural equation modeling. A consensus on the ideal sample size for factor analysis and structural models has yet to be available. The dependability of the measures, factors, and items utilized could influence the sample size for a CFA. To determine the CFA sample size, follow well-established guidelines. According to these guidelines, it is recommended to maintain a ratio of 10 to 15 subjects per variable or ensure a minimum of 5 cases per variable [26]. They are flexible, and the determination of sample size can be affected by various factors, including the characteristics of the data and the measurement model. In order to confirm the structure of the variables in the model, we needed 150 samples. The final sample size was 180 individuals after accounting for the possibility of non-responses (20%).

### Study period and measurement data

The survey was conducted from November to December 2021. The tool used in the study was a questionnaire measuring ICS with 30 items [19]. A total of seven factors were examined in this study: general (6 items), hearing (4 items), saying (4 items), asking (4 items), clarifying (4 items), encouraging (4 items), and feedback (4 items). We collected responses on a five-point Likert scale ranging from “very high” to “very low.“

### Data collection method

In the first step, a list of urban and rural CHCs was determined. In the next step, we determined the sample proportion for each center. Researchers were chosen based on easy-to-access multi-level sampling criteria. Three rural and three urban CHCs were included in the study, each accounting for 53% and 47% of the sample. The sample included a diverse range of health professionals and employees, such as doctors, nurses, midwives, laboratory technicians, health services staff, and administrators. A team of skilled investigators was enlisted to help distribute surveys. In addition to supervision by researchers with expertise in scale development, data collectors were extensively trained in proper implementation, ethics, data collection methods, cultural sensitivity, and careful monitoring of data quality. In approximately 30 min, participants responded to the tool. Fortunately, less than 1% of respondents declined to answer the survey. A verbal incentive-based approach has been successful in increasing survey participation, such as “By participating in this questionnaire, we will be better able to meet your needs.“ Email was used as a contact method to invite participants to take part in the research study and follow-up.

### Methods of analysis

The descriptive statistics were calculated and performed. All missing values were replaced with their averages. The components were then extracted using Lisrel 8.8 [27] and Smartpls 3.2.8 [28]. For the validation of the ICS, first- and second-order CFA were used. The fit indexes used to assess the model’s fit with the data were the GFI, the AGFI, the CFI, the NFI, the SRMR, the RMSEA, and relative chi-square statistic (χ2/DF) [29]. In assessing model fit, it is essential to take into account several fit measures instead of relying on one. A model’s fit to data can be better understood if researchers examine a variety of metrics. These indices must also be interpreted flexibly and not as rigid yes/no statements. In order to assess the discriminant validity of the ICS, the Fornell-Larcker criterion was applied [30]. To establish discriminant validity, we need to compare the AVE [31] value with other variables’ correlation values. The correlation coefficient between the item and one of its components must also be meaningfully higher. Cronbach’s alpha measured ICS’ internal reliability, CR [32], and intra-cluster correlation coefficients (ICC) [33].

### Ethical clearance

After receiving authorization from the scale developer, the AJUMS Ethics Committee approved the study (IR. AJUMS.REC.1399.633). The Research team introduced the research objectives to the participants and stressed that their involvement was voluntary. All participants gave consent for participation, and data were collected anonymously.

## Results

### Characteristics of the participants

There were 170 participants in this study, and 0.06% of them did not respond. Study participants’ sociodemographic characteristics are described in Table 1. The age range was 22–61 years, with an average age of 36.68 years and a standard deviation of 7.52 years.

**Table 1:** Demographic characteristics of healthcare workers (n = 170)

Variable		Frequency(Percent)
Gender	Femal	119(70)
	Male	51(30)
Marital status	Single	50(30)
	Married	120(70)
Education	< licence	73(43)
	>= licence	97(57)
History_job	< 10 year	85(50)
	>= 10 year	85(50)
Place	Urban	89(52.3)
	Rural	81(47.7)

### Analyzing items

For the purpose of validating the EICS, we employed both first- and second-order CFAs. In terms of skewness, the values ranged from − 3.323 to -0.600, and in terms of kurtosis, they ranged from 0.109 to 16.408. The normalized kurtosis coefficient (Mardia) was less than 5, indicating deviation from multivariate normality. A weighted least squares estimation was used to reduce the influence of outliers and minimize the impact of non-normal data.

### Analyzing reliability

Cronbach’s alpha coefficients, CR, and ICC confirmed the EICS’ internal validity (Table 2).

**Table 2:** Construct Reliability and Validity of the ICSS (n = 170)

ICSS	Mean [95% CI]	Std. Dev [Std. Err]	AVE	alpha	CR	ICC [95% CI]	n
General	4.53 [4.47 to 4.59]	0.47 [0.030]	0.46	0.81	0.83	0.81 [0.77 to 0.84]	6
Say	4.55 [4.48 to 4.62]	0.53 [0.033]	0.58	0.84	0.85	0.84 [0.80 to 0.87]	4
Hear	4.53 [4.46 to 4.60]	0.55 [0.035]	0.68	0.89	0.89	0.89 [0.87 to 0.91]	4
Clear	4.13 [4.04 to 4.22]	0.70 [0.045]	0.54	0.75	0.82	0.75 [0.70 to 0.80]	4
Ask	4.16 [4.08 to 4.25]	0.68 [0.044]	0.58	0.85	0.85	0.85 [0.81 to 0.88]	4
Feedback	4.36 [4.29 to 4.32]	0.57 [0.037]	0.64	0.87	0.88	0.87 [0.85 to 0.90]	4
Admire	4.41 [4.33 to 4.48]	0.58 [0.038]	0.58	0.84	0.84	0.84 [0.80 to 0.87]	4

### Analyzing validity

The first-order CFA results showed that all indicators had reasonable t-values and factor loadings. In view of the extracted root values of AVE, it confirms the first-order CFA model by demonstrating adequate divergent validity by correlating each component with other components (Table 3). Furthermore, GFI and CFI exceeded 0.8 (χ2 = 767.17; DF = 375; P < 0.001; CFI = 0.98; GFI = 0.82; AGFI = 0.80; NFI = 0.97; SRMR = 0.22; RMSEA = 0.068) values indicating a good fit for the model (Table 4).

**Table 3:** Discriminant validity of the ICSS (Fornell—Larcker criterion)

Subscales	Hear	Feedback	Say	Ask	Admire	Clear	General
Hear	0.83						
Feedback	0.72	0.80					
Say	0.83	0.66	0.76				
Ask	0.72	0.73	0.63	0.76			
Admire	0.76	0.73	0.73	0.69	0.76		
Clear	0.74	0.74	0.72	0.69	0.67	0.74	
General	0.72	0.56	0.72	0.53	0.61	0.61	0.68

**Table 4:** First-order CFA of the ICSS (n = 170)

Factors							
Items	**I**	**II**	**III**	**IV**	**V**	**VI**	**VII**
**I** = General Q1 Q2 Q3 Q4 Q5 Q6	0.73* 0.73* 0.68* 0.84* 0.83* 0.87*						
**II** = Say Q1 Q2 Q3 Q4		0.93* 0.96* 0.64* 0.82*					
**III** = Hear Q1 Q2 Q3 Q4			0.89* 0.90* 0.84* 0.85*				
**IV** = Clear Q1 Q2 Q3 Q4				0.78* 0.70* 0.75* 0.81*			
**V =** Ask Q1 Q2 Q3 Q4					0.72* 0.71* 0.84* 0.52*		
**VI =** Feedback Q1 Q2 Q3 Q4						0.79* 0.85* 0.82* 0.88*	
**VII =** Admire Q1 Q2 Q3 Q4							0.81* 0.89* 0.87* 0.70*

χ2 = 767.17; DF = 375; P < 0.001; CFI = 0.98; GFI = 0.82; AGFI = 0.80; NFI = 0.97; SRMR = 0.22;

CN = 127.83; RMSEA = 0.068. * T-value > 3.54(P-value < 0.001**)**

The second-order CFA results showed that all indicators had reasonable t-values and factor loadings between seven confirmed first-order CFA model correlations and the ultimate factor(Table 5). In this phase, we assessed the accuracy of the measurement of the EICS subscales. (χ2 = 797.24; DF = 381; P < 0.001; CFI = 0.98; GFI = 0.82; AGFI = 0.78; NFI = 0.97; SRMR = 0.059; RMSEA = 0.068)

**Table 5:** s-order CFA of the ICSS (n = 170)

Factors							
Items	**I**	**II**	**III**	**IV**	**V**	**VI**	**VII**
**I** = General Q1 Q2 Q3 Q4 Q5 Q6	0.79* 0.74* 0.75* 0.82* 0.81* 0.84*						
**II** = Say Q1 Q2 Q3 Q4		0.86* 0.89* 0.65* 0.70*					
**III** = Hear Q1 Q2 Q3 Q4			0.81* 0.86* 0.78* 0.78*				
**IV** = Clear Q1 Q2 Q3 Q4				0.77* 0.67* 0.68* 0.74*			
**V =** Ask Q1 Q2 Q3 Q4					0.74* 0.78* 0.79* 0.75*		
**VI =** Feedback Q1 Q2 Q3 Q4						0.75* 0.82* 0.79* 0.84*	
**VII =** Admire Q1 Q2 Q3 Q4							0.71* 0.86* 0.83* 0.64*

χ2 = 797.24; DF = 381; P < 0.001; CFI = 0.98; GFI = 0.82; AGFI = 0.78; NFI = 0.97; SRMR = 0.059;

CN = 127.33; RMSEA = 0.068. * T-value > 3.54(P-value < 0.001)

Using the Fornell-Larcker matrix, we assessed the model’s discriminant validity.

### Identifying priorities for effective decision-making

**Fig. 1:**
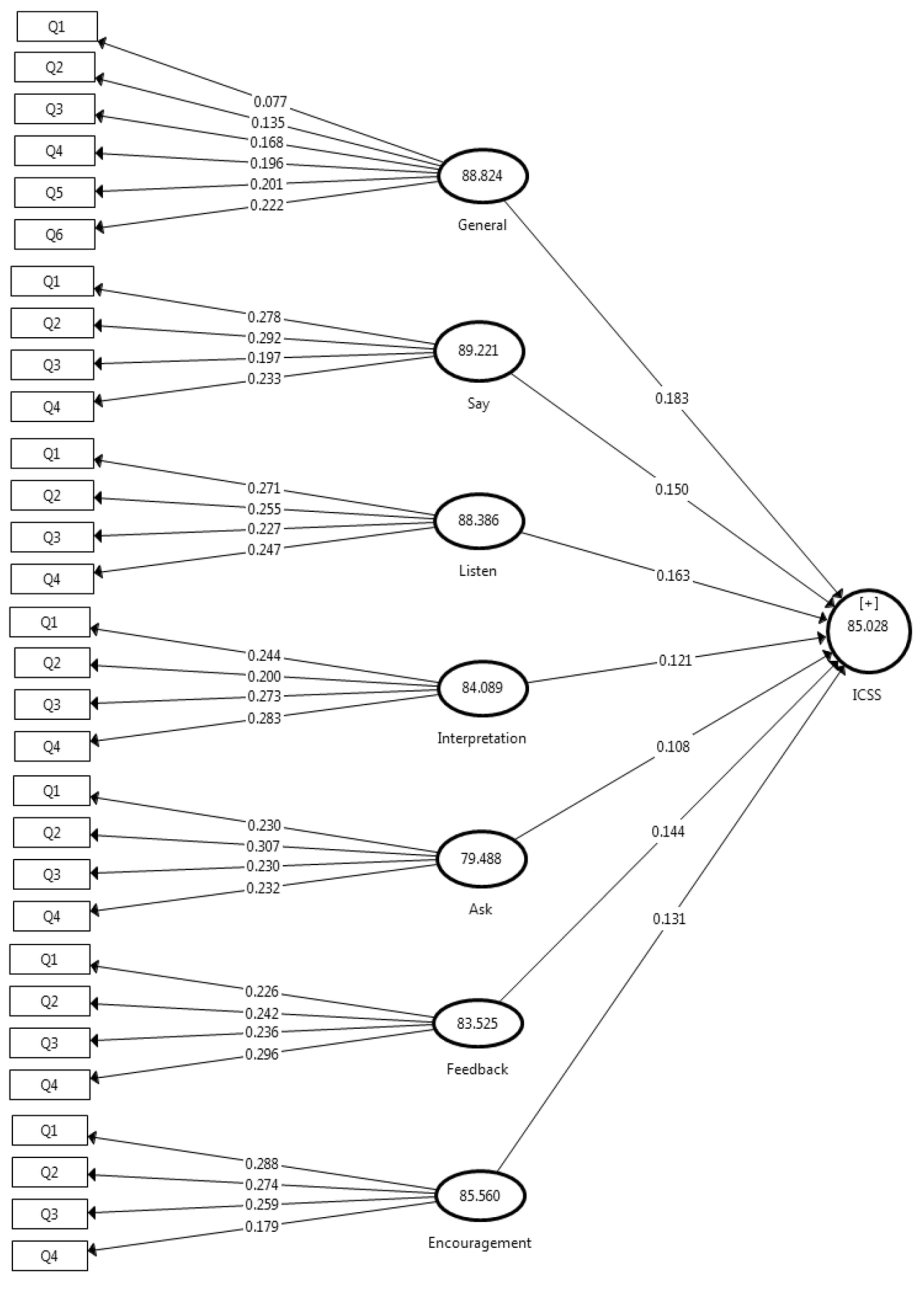
The importance-performance map analysis of constructs on the conceptual model

The Importance Performance Map Analysis (IPMA) [34] is a valuable technique for examining the influence of constructs within a conceptual model (Fig. 1). In this study, we employed IPMA in partial least squares structural equation modeling with EICS as the primary variable. In terms of IPMA, general and saying skills received the highest scores while asking and clarifying skills scored the lowest.

## Discussion

Communication skills in HCPs often need more refinement due to inadequate education and a lack of understanding of the importance of providing services. Studies have shown that improving communication skills can enhance client satisfaction in healthcare settings [35, 36].

Our study aimed to evaluate EICS among urban and rural CHCs of AJUMS staff. In the study, Compared to other communication skills, the mean [95% CI] scores for general and saying were higher. While the mean [95% CI] scores for clarifying and asking were lower. In the Siamian et al. study, the highest score was observed in the “Punishing and Encouraging Skills,“ while the “Feedback” skill received the lowest score. They indicated that public relations skills, listening, reward and punishment were well-developed, whereas other skills were rated as average [37].

However, there is yet to be a universal approach to assessing communication skills across different fields. It has been noted that most communication assessment instruments still need to be validated [38]. We are not recommending a specific assessment instrument. However, a multidimensional approach can be employed to consider various aspects of communication. In this study, the original seven-factor structure of the EICS was supported, consistent with other studies [19]. The comprehensive structure of EICS sets it apart from other instruments designed to assess ICS in health profession education. In a healthcare setting, effective and meaningful communication is crucial for delivering high-quality patient care [39]. Effective communication is an essential element for providing quality care in the healthcare system and improving educational quality [40]. EICS should be regarded as a significant prerequisite in educational processes. We can enhance EICS by seeking feedback, actively listening, using positive body language, managing emotions, acknowledging others, and practicing active listening to build stronger relationships. The seven-factor model offers can serve as a valuable tool for educators and researchers in health professions, distinguishing itself from existing instruments [41].

Our results indicated that the seven-factor solution was appropriate for recognizing the components of the EICS. Different studies have proposed various models with different numbers of factors or subscales. The Ghasemi et al. (2014) identified six factors [20]. Furthermore, some researchers have shown interest in determining the best-fitting model through exploratory factor analysis (EFA) or CFA and comparing alternative models. Some studies support a unidimensional structure, while others propose multidimensional models with factors such as listening skills, assertiveness, or nonverbal communication [42]. In this research, in addition to simplifying the EICS, one factor was added. The inclusion of another factor suggests that additional resources play a role in effective communication procedures. By considering it, researchers can gain insights into the specific resources needed to tailor communication strategies to different segments of the target population. This understanding can lead to more effective and targeted communication interventions, ultimately enhancing health behavior outcomes.

The CFA results indicated that the measurement model was robust and provided an excellent fit to the data. It is due to the indicators’ validity, divergent validity, model fit indicators, second-order CFA, discriminant validity, and chi-square statistic. Indicator validity is confirmed through the t-values and factor loadings of all indicators in both first-order and second-order CFA.

Divergent validity was confirmed through the extracted root values of AVE, indicating that each construct was sufficiently distinct from others. Model fit indicators, such as the GFI and CFI, were positive signs that the proposed model fits the observed data well.

Second-order CFA successfully implemented suggests that the higher-order factor effectively captures the shared variance among the first-order factors, providing a more parsimonious representation of the relationships among latent constructs.

Discriminant validity was confirmed using the Fornell-Larcker matrix, ensuring that the constructs were distinct from each other.

The significant chi-square statistic is a common finding, especially in larger samples, but it should be interpreted cautiously considering other fit indices.

The reliability and validity of the measurement model were crucial for researchers and practitioners to make meaningful interpretations and draw accurate conclusions based on the collected data.

Limitations and future research should be acknowledged, such as sample characteristics, data collection methods, or specific assumptions made in the CFA. In conclusion, the reported findings suggest that the measurement model used in the study was robust and valid, enhancing the credibility of the study and contributing to the overall understanding of the relationships among variables under investigation.

In terms of IPMA, the results of our analysis revealed that general and listening skills obtained the highest scores. It indicates that these skills are both highly significant and well-performed in the context of our study. These findings have important implications for relevant settings, as focusing on and enhancing these skills could yield positive outcomes and improve communication effectiveness. In contrast, the study found that asking and interpretation skills in Iran are less critical than in other cultures due to cultural differences, communication styles, the Iranian educational system, workplace dynamics, and language nuances. Cultural norms may prioritize implicit communication or contextual understanding over direct questioning and interpretation, which could explain the lower importance assigned to these skills. Communication styles also play a role, with some cultures favoring listening over asking questions and relying on contextual cues for interpretation. The Iranian educational system may place less emphasis on teaching or assessing these skills, leading to a lower perceived importance. The specific context of the study, such as work or social interactions, could also influence the importance assigned to specific skills. Finally, the Iranian language and communication norms may have nuances that make asking and interpretation skills less prominent, making them less prominent. Further research is needed to understand the specific cultural and contextual reasons behind the observed lower scores for asking and interpretation skills in the Iranian context.

Conversely, low scores for questioning and interpretation skills indicate potential areas for improvement. Identifying the reasons behind their lower performance and understanding their impact on EICS can provide valuable insights. It may be worth exploring strategies to enhance these skills or identifying any contextual factors that hinder their effectiveness.

**Fig. 2:**
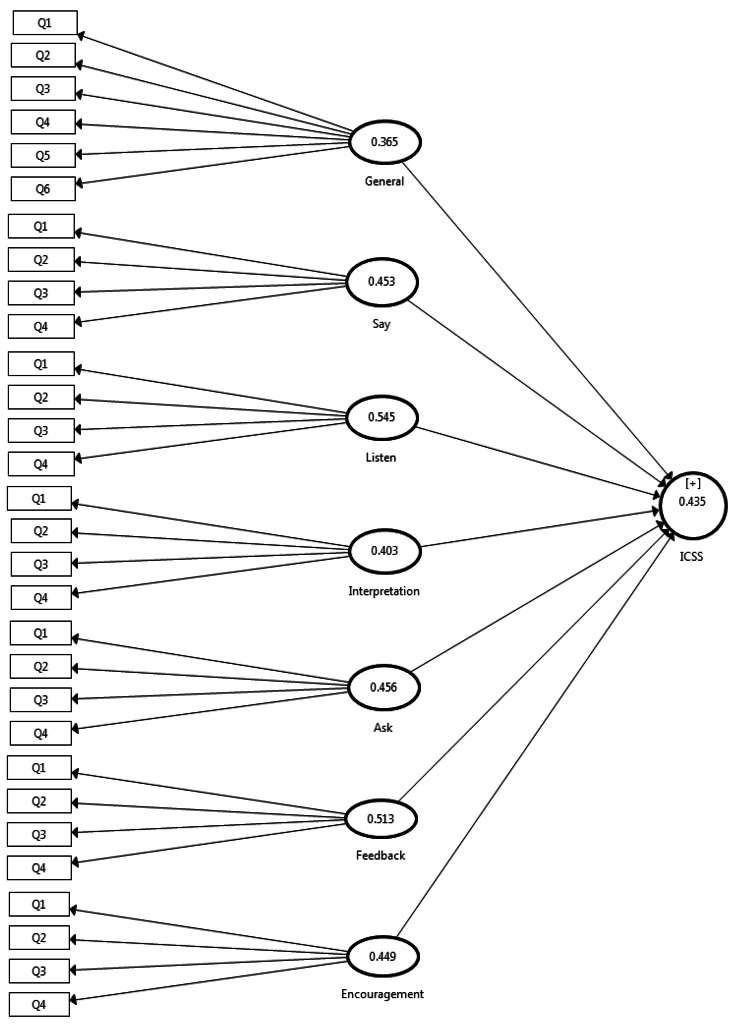
The predictive power of the conceptual model using the Q2-index

The predictive power of the current model, based on the blindfolding test (Q2-index) [43], was determined to be 44% (Fig. 2). Since we have identified the seven factors in EICS as a fundamental need for HCPs, it is necessary to consider the importance of learning and applying these skills in their retraining programs. Unfortunately, no studies have conducted CFA to validate the EICS among HCPs. We cannot compare our findings with other studies. Nevertheless, the reliability findings of the EICS were consistent with most studies conducted in communication skill assessment [44]. Some studies demonstrated high internal consistency and test-retest reliability, while others raised concerns about specific items or subscales. In addition, the validity of ICS has been examined through its correlation with related measures, such as communication competence or social interaction skills [45].

ICS maintains a stable factor structure that can be reliably assessed using both EFA and CFA. The measurement invariance analysis further confirmed that the scale measures ICS consistently across cultures. Further evaluations of this model will be necessary to compare the results. EICS can be influenced by cultural factors such as language, belief systems, morality, and perspective. Additionally, personal and family characteristics can play a significant role in shaping EICS.

These results suggest that EICS is a helpful tool for assessing comprehensive HCPs in Iran. A comprehensive validation process can be carried out with EICS, which started with this study and will continue. This validation process was rigorous, and it permits others to evaluate it in a variety of contexts.

### Limitations of the study

There is a concern that the findings from the University of Medical Sciences in Iran cannot be generalized to other universities. In any study, generalizability depends on a variety of factors, such as the research design, the sample size, and the characteristics of the studied population. When interpreting the findings of any study, it is crucial to consider its context and limitations. This study highlights the limitations of self-report measures in accurately reflecting respondents’ experiences with their EICS. Alternative data collection methods are needed due to social desirability bias and inaccurate recall, which lead to unreliable data.

## Conclusions

The psychometric properties of effective interpersonal communication skills, as evidenced by their reliability, conciseness, and validity, establish it as a valuable tool for them. Its utilization empowers Healthcare Professionals to make well-informed decisions regarding the health and treatment of their clients. This scale holds the potential to serve as a foundation for the development of educational programs within healthcare professional education. To evaluate this prospect, researchers may consider employing datasets sourced from diverse medical science universities globally.

## Data Availability

Materials and data are available upon request from Dr. Hashem Mohamadian.

## Abbreviations

AGFI: Adjusted Goodness of Fit
AJUMS: Ahvaz Jundishapur University of Medical Sciences
AVE: Average Extracted Variable
CHCs: Comprehensive Health Centers
CFA: Confirmatory Factor Analysis
CFI: Comparative Fit Index
CR: Construct Reliability
HCPs: Healthcare Professionals
EICS: Effective Interpersonal Communication Skills
ICS: Interpersonal Communication Skills
ICC: Intra-cluster Correlation Coefficients
IPMA: Importance-Performance Map Analysis
GFI: Goodness of Fit Index
RMSEA: Root Mean Square Error of Approximation
SRMR: Standardized Root Mean Square Residual
